# Population-based study of effectiveness of neoadjuvant radiotherapy on survival in US rectal cancer patients according to age

**DOI:** 10.1038/s41598-017-02992-7

**Published:** 2017-06-14

**Authors:** Leilei Wu, Shichao Pang, Qianlan Yao, Chen Jian, Ping Lin, Fangyoumin Feng, Hong Li, Yixue Li

**Affiliations:** 1 School of Life Sciences and Biotechnology, Shanghai Jiao Tong University, Shanghai, 200240 China; 2 CAS Key Laboratory for Computational Biology, CAS-MPG Partner Institute for Computational Biology, Shanghai Institute for Biological Sciences, Chinese Academy of Sciences, Shanghai, 200031 China; 3 Collaborative Innovation Center of Genetics and Development, Fudan University, Shanghai, 200433 China; 4 Department of Statistics, School of Mathematical Sciences, Shanghai Jiao Tong University, Shanghai, 200240 China; 5 Department of General Surgery, Shanghai General Hospital, Shanghai Jiao Tong University School of Medicine, Songjiang District, 201600 shanghai, China; 6 School of Life Science and Technology, ShanghaiTech University, Shanghai, 201210 China

## Abstract

Recent cancer researches pay more attention to younger patients due to the variable treatment response among different age groups. Here we investigated the effectiveness of neoadjuvant radiation on the survival of younger and older patients in stage II/III rectal cancer. Data was obtained from Surveillance, Epidemiology, and End Results (SEER) database (n = 12801). Propensity score matching was used to balance baseline covariates according to the status of neoadjuvant radiation. Our results showed that neoadjuvant radiation had better survival benefit (Log-rank *P* = 3.25e-06) and improved cancer-specific 3-year (87.6%; 95% CI: 86.4–88.7% vs. 84.1%; 95% CI: 82.8–85.3%) and 5-year survival rates (78.1%; 95% CI: 76.2–80.1% vs. 77%; 95% CI: 75.3–78.8%). In older groups (>50), neoadjuvant radiation was associated with survival benefits in stage II (HR: 0.741; 95% CI: 0.599–0.916; *P* = 5.80e-3) and stage III (HR: 0.656; 95% CI 0.564–0.764; *P* = 5.26e-08). Interestingly, neoadjuvant radiation did not increase survival rate in younger patients (< = 50) both in stage II (HR: 2.014; 95% CI: 0.9032–4.490; *P* = 0.087) and stage III (HR: 1.168; 95% CI: 0.829–1.646; *P* = 0.372). Additionally, neoadjuvant radiation significantly decreased the cancer-specific mortality in older patients, but increased mortality in younger patients. Our results provided new insights on the neoadjuvant radiation in rectal cancer, especially for the younger patients.

## Introduction

Colorectal cancer (CRC) is the third leading cause of cancer-related death in the US. About a third of newly diagnosed CRC patients develop from the rectum, and others develop from the colon^1^. The genomes of some CRC patients are hypermutated and three quarters of these have highly microsatellite instability (MSI). Among the non-hypermutated tumors, colon and rectum cancers have similar patterns of genomic landscape^2^. Large-scale genome studies have revealed significantly intra-tumor heterogeneity and recurrent mutated genes in rectal cancer, such as *APC*, *TP53*, *KRAS* ^2, 3^. Additionally, many biomarkers such as Astrocyte elevated gene-1 (*AEG-1*)^4^, *CD163* ^5^ and clinical factors such as age^6^, stage^7^ have been proven to be associated with rectal cancer prognosis. Rectal cancer has higher risk of local recurrence than colon cancer. Therefore, multimodal therapy is required for treatment of rectal cancer.

Patients with rectal cancer that have not spread to distant sites are usually treated with surgery and additional treatment with radiation and chemotherapy may be used before or after surgery. The goal of using adjuvant radiation therapy for rectal cancer is to prevent local recurrence and mortality in patients with locally advanced tumors. Postoperative radiation therapy alone only improves local control; Additional concurrent chemotherapy to post-radiotherapy not only reduces local recurrent rate but also improves survival^8^. In 1990, US National Institutes for Health recommended postoperative chemoradiotherapy as standard treatment for the completely resected stage II or III rectal cancer patients in the US^9^. However, the treatment of rectal cancer has been changed over time with postoperative radiotherapy decreasing from 25% to 4% (1980–1988 vs. 1995–2000), while preoperative radiotherapy increasing from 1 to 35% (1980–1988 vs. 1995–2000)^10^. In 1994, European Consensus Conference recommended preoperative radiotherapy for patients with T3/ T4 and/or node-positive cancer^11^. Another recent study also shows that a significantly increase in the use of preoperative radiotherapy for rectal cancer from 1998 to 2007^12^. Many large-scale studies prove that preoperative radiotherapy reduces the rate of local recurrence^13, 14^ and improves overall survival^15^. Compared with postoperative chemoradiotherapy, preoperative radiotherapy has better local control^16^, is associated with reduced toxicity^14^ and is a more effective treatment^17^.

However, previous studies did not compare the effectiveness of neoadjuvant radiotherapy between subgroups. Clinical factors such as age^6^ and stage^7^ have been proven to be associated with rectal cancer prognosis. Therefore, important prognostic clinical factors are often used to stratify patients. For example, in age aspect, CRC screening standard is recommended for adults above 50 years since 1996^18^. Recently studies find the incidence rate of CRC among adults younger than 50 years is increasing^19, 20^ which is in sharp contrast to overall incidence trend among adults above 50 years^21^. Additionally, different prognostic molecular biomarkers such as *PRL*, *RBM3* in younger patients^6^, clinical risk factors such as varied distribution of stage, histological type, grade^19, 22, 23^ and different responses to the same treatment^24^ have been shown between two age subgroups. Therefore, it is important to investigate the effectiveness of neoadjuvant radiation in different subgroups.

Survival rate is commonly used to determine the treatment advances. To estimate survival rate, clinical characteristics are recoded and analyzed in randomized trials^14, 25^ or in population based studies^26^. The public database such as Surveillance, Epidemiology, and End Results (SEER) provides clinical data for different cancer cases, which is a valuable resource for survival analysis. Since SEER is a population-based cancer registry, controlling treatment selection bias can get a more reliable results^27^. Recently propensity score methods have been widely used to adjust baseline covariates, which can control the treatment selection bias. Propensity score is the probability of a subject receiving the treatment of interest conditional on their observed baseline covariates^28^. Through propensity score matching, 98% of the treatment bias are removed and an unbiased estimate of treatment effect is achieved in retrospective cohorts^29^.

Here we aimed to investigate the effectiveness of neoadjuvant radiation in younger and older rectal cancer patients. Clinical data was obtained from SEER, and propensity score matching was used to balance patients with or without neoadjuvant radiation. The effect of neoadjuvant radiation on survival was analyzed among patients mainly stratified by age at diagnosis. Our results might reinforce the consideration of younger patients when receiving neoadjuvant radiation.

## Method

### Data preprocessing

We signed an agreement for the SEER 1973–2012 research data and all clinical data were approved to use by SEER database. Then we downloaded the classified data of colorectal cancers from SEER database (1973~2012), and used R package SAScii to decode the original data into clinical information. In all CRC patients, we restricted our analysis to rectal cancer patients whose death was caused by cancer, date of diagnosis was after 2007, tumor grade was stage II or III, and treatment strategy was surgery alone or radiotherapy before surgery.

### Statistical analysis

#### Propensity score matching

We defined propensity score as the probability of patients being in the neoadjuvant radiation group. Propensity score model is preferable to include prognostically important covariates than these that affecting the treatment-selection process^30^. Firstly, we select these important prognostic factors: tumor size^31^, histological type^32^, differentiation^31^, age^6^, number of harvest lymph node^33, 34^, tumor stage^7^. Secondly, some confounding variables which included in previous CRC^6, 32, 35^ or propensity score based research^27^ and might be related with treatment and outcome were selected. Finally, in combination with records in SEER, nine covariates: race, gender, age, tumor size, tumor number, stage, histological type, grade, lymph node resected were used to build propensity score models. The propensity scores were calculated by a nonparsimonious multivariable logistic regression model, which used neoadjuvant radiotherapy as the outcome of interest and nine clinical features as covariates. A nearest neighbor and 1 to 1 matching algorithm was performed within default caliper (0.2) in SPSS^36^. Standardized difference (S.D) between neoadjuvant radiation and no-neoadjuvant radiation patients was calculated for every baseline covariate to test corresponding balance^29^. S.D before and after matching was compared to see whether the S.D was changed to less than 0.1, which indicated a good balance for matched data^30^. A sensitivity analysis was performed to test the robustness of matched data using the R package rbounds^37^. R packages powerSurvEpi^38, 39^ was used to estimate the sample size necessary for 80% statistically power.

#### Cancer specific survival curve and Cox model construction

In propensity score matched SEER dataset, survival time was calculated from the date of diagnosis to death or to the end of study. Patients who were lost followed-up or still alive at the end of study were censored. Cancer-specific survival curve was generated using Kaplan–Meier estimate, and difference between different treatment groups were analyzed by log-rank test. Additionally, 3-year and 5-year cancer specific survival rates were calculated for neoadjuvant radiation and no-neoadjuvant radiation groups. Cox proportional hazards regression model which contained all measured covariates was performed and hazard ratio (HR) was calculated to assess the importance of neoadjuvant radiotherapy and other covariates. To assess the effects of unmeasured clinical variables on HR, sensitivity analysis^40–42^ was performed by R package obsSens.

All Survival rate, HR and risk ratio (RR) were reported with their corresponding 95% CI. All statistical test were two-sided and a *P* value of less 0.05 was considered to be statistically significant.

#### Patient stratification by clinical covariates

Based on the results of Cox model, we selected clinical covariates associated with neoadjuvant radiotherapy. We grouped patients by each covariate, performed cancer specific survival analysis and constructed Cox model in each subgroup.

#### Effect of age on neoadjuvant treatment analysis

We divided patients into younger and older groups. For each group, annual age-adjusted incidence rates (using the 2000 U.S. standard population) were calculated of rectal cancer per 100,000 from 1992 to 2012 using SEER*Stat software version 8.3.4^43, 44^. Fisher’s exact test was used to compare the distribution of every clinical variable item between younger and older patients. Cancer specific survival curve and Cox proportional hazards regression were performed to evaluate the effectiveness of neoadjuvant radiotherapy. HR was calculated to assess the importance of neoadjuvant radiotherapy and also 3, 5 year survival rates were calculated in different treatment groups. The RR was calculated in different time interval for younger and older groups. We further combined age and stage to stratify patients, and investigate the effectiveness of neoadjuvant radiotherapy in four subgroups.

## Results

### Data source and propensity score matching

**Figure 1:**
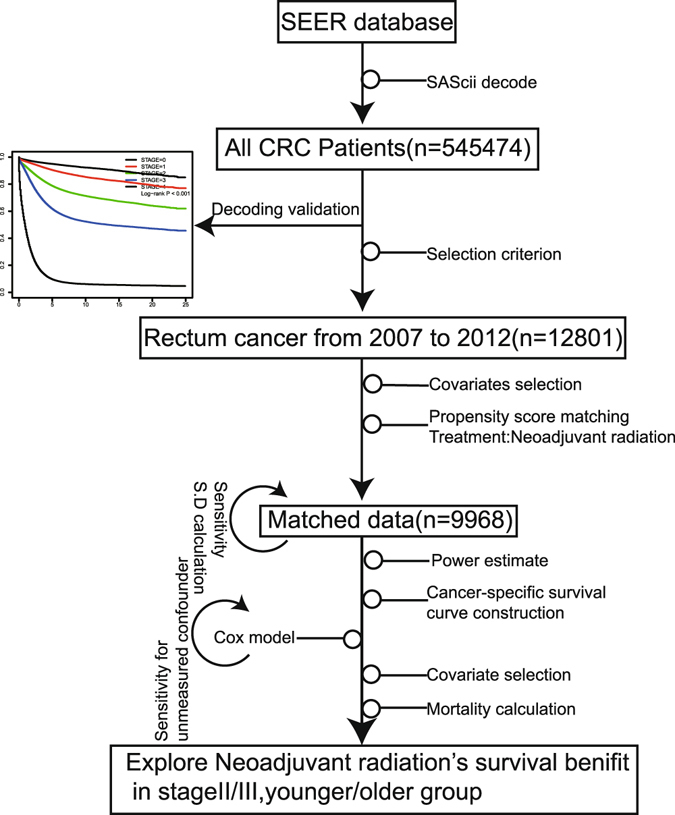
The workflow of comprehensively access the impact of neoadjuvant radiation on survival in rectal cancer patients.

We performed a comprehensive study to access the impact of neoadjuvant radiation on survival in rectal cancer patients (Fig. 1). Firstly, clinical data of 545474 CRC patients were obtained from the SEER database. Stage-specific survivals were significantly different in five stages, which was consistent with a previous study^7^. Adults over age 50 are high-risk population of CRC who are recommended to receive CRC screening. Our data also showed that 80% rectal patients were above age 50 (see Supplementary Fig. 1AB). We also calculated annual age-adjusted incidence rate per 100,000 between younger and older groups for rectal cancer during 1992~2012. Trend of incidence rate decreased in adults over age 50, while rose for adults less 50 (see Supplementary Fig. 1C), which was consistent with the trend in previous CRC studies^19, 20^. Therefore, age 50 was used to divide patients into younger (age < = 50, n = 2511) and older groups (age >50, n = 10290). Totally, There were 12801 rectal patients in stage II or III, or whom 6117 (48.25%) had neoadjuvant radiation before surgery since 2007. Their clinical characteristics were summarized in Table 1.

**Table 1:** Comparison of clinical characteristics between patients with or without neoadjuvant radiotherapy in the original dataset and in the propensity score matched dataset.

Clinical Characters	Before Propensity Score Matching			After Propensity Score Matching		
	Sn (%)	Sn + PRT (%)	SD	Sn (%)	Sn + PRT (%)	SD
**RACE**						
Withe	5304 (80.07)	4997 (80.90)	−0.0208	3996 (80.18)	3997 (80.20)	−0.0005
Black	596 (9.00)	504 (8.16)	0.0299	461 (9.25)	427 (8.57)	0.0239
Unknown	724 (10.93)	676 (10.94)	−0.0004	527 (10.57)	560 (11.24)	−0.0212
**GENDER**						
Male	3576 (53.99)	3868 (62.62)	−0.1758	2961 (59.41)	3026 (60.71)	−0.0266
Female	3048 (46.01)	2309 (37.38)		2023 (40.59)	1958 (39.29)	
**AGE**						
< = 50	956 (14.43)	1555 (25.17)	−0.2720	956 (19.18)	1027 (20.61)	−0.0357
>50	5668 (85.57)	4622 (74.83)		4028 (80.82)	3957 (79.39)	
**TUMOR NUMBER**						
Single	6046 (91.27)	5757 (93.20)	−0.0720	4588 (92.05)	4608 (92.46)	−0.0150
Multiple	578 (8.73)	420 (6.80)		396 (7.95)	376 (7.54)	
**TUMOR SIZE**						
< = 5 cm	4250 (64.16)	4518 (73.14)	−0.1945	3467 (69.56)	3465 (69.52)	0.0009
>5 cm	2374 (35.84)	1659 (26.86)		1517 (30.44)	1519 (30.48)	
**TNM STAGE**						
II	2903 (43.83)	2647 (42.85)	0.0196	2133 (42.80)	2131 (42.76)	0.0008
III	3721 (56.17)	3530 (57.15)		2851 (57.20)	2853 (57.24)	
**HISTOLOGICAL TYPE**						
Non-mucinous	6234 (94.11)	5686 (92.05)	0.0813	4658 (93.46)	4585 (91.99)	0.0564
Mucinous	353 (5.33)	449 (7.27)	−0.0799	291 (5.84)	373 (7.48)	−0.0660
SRCC	37 (0.56)	42 (0.68)	−0.0155	35 (0.70)	26 (0.52)	0.0232
**GRADE**						
Well	358 (5.40)	378 (6.12)	−0.0307	291 (5.84)	340 (6.82)	−0.0404
Moderately	5166 (77.99)	4878 (78.97)	−0.0239	3917 (78.59)	3881 (77.87)	0.0175
Poorly	992 (14.98)	827 (13.39)	0.0455	703 (14.11)	686 (13.76)	0.0098
undifferentiated	108 (1.63)	94 (1.52)	0.0087	73 (1.46)	77 (1.54)	−0.0066
**LYMPH NODE**						
negative	91 (1.37)	310 (5.02)	−0.2083	91 (1.83)	168 (3.37)	−0.0972
< = 12	1224 (18.48)	2127 (34.43)	−0.3678	1181 (23.70)	1360 (27.29)	−0.0825
>12	5309 (80.15)	3740 (60.55)	0.4394	3712 (74.48)	3456 (69.34)	0.1145

Sn: surgery only; Sn + PRT: pre-radiation treatment + surgery; SD: standardized difference; SRCC: sing-ring cell carcinoma.

There were systematic differences in baseline characteristics between neoadjuvant radiation and no-neoadjuvant radiation patients in the overall samples. The S.Ds of gender, age, lymph node resected etc were larger than 0.1 between neoadjuvant radiation and no-neoadjuvant radiation patients which means the distribution of those items were unbalanced (Table 1 **left**). Therefore, a propensity score model was built to get a similar distribution of these measured baseline covariates. After propensity score matching, the standardized differences of all covariates were less than or close to 0.1 (Table 1 **right**), which indicated the covariates were balanced between two treatment groups. This balanced cohort totally had 9968 (77.8%) patients, and the number of two group patients were same (n = 4984). Furthermore, we did the sensitivity analysis to test whether the matched dataset was robust to clinical variables that were not considered in the propensity score model. The results showed our matched data was robust to the unconfouned variants (Γ = 7, *P* = 2.1e-05), as described in previous studies^37^. To determine whether the sample size was enough to reach a power of 0.8, we estimated the number of patients for survival analysis and the number of deaths for Cox model. Result showed that at least 1096 patients and 302 deaths were needed in each treatment subgroup. Therefore, our propensity score matched dataset (4984 in each group) could achieve higher power.

### Cancer-specific survival analysis and Sensitivity analysis

#### Survival benefit of neoadjuvant radiation

**Figure 2:**
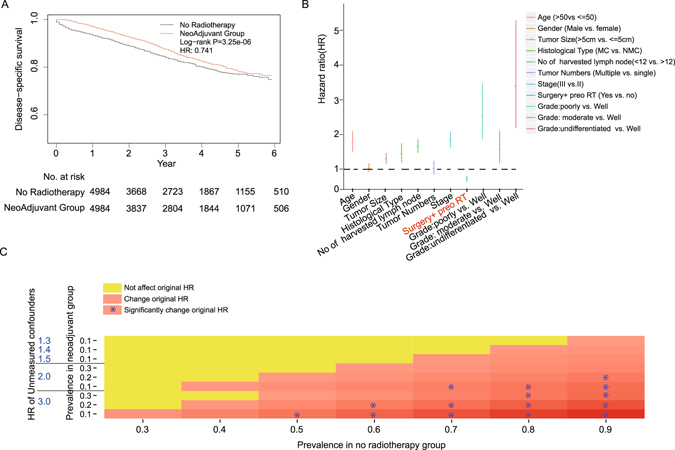
Comparison between neoadjuvant (n = 4984) and no-neoadjuvant groups (n = 4984) in the propensity score matched cohort. (**A**) Kaplan-Meier curves for cancer-specific survival. (**B**) HR of every clinical covariate from Cox model. (**C**) Sensitivity analysis estimating the effect of unmeasured confounders on hazard ratio. Red star means significantly render treatment effect (the lower confidence bound of CI is larger than 1).

In the propensity score matched cohort, the median follow-up time was 27 months (ranged 0–71 months). The 3-year and 5-year cancer-specific survival (CSS) rates were 87.6%(95% CI: 86.4–88.7%), 78.1%(95% CI: 76.2–80.1%) in neoadjuvant radiation group and 84.1%(95% CI: 82.8–85.3%), 77%(95% CI: 75.3–78.8%) in no-neoadjuvant radiation group. The CSS rates of neoadjuvant radiation group was significantly better than no-neoadjuvant group (Fig. 2A, Log-rank *P* = 3.25e-06). The neoadjuvant radiotherapy was associated with reduced disease-specific mortality at 6 months (RR, 0.25; 95% CI, 0.18–0.35), 1 year (RR, 0.44; 95% CI, 0.35–0.55) and 2 year (RR, 0.59; 95% CI, 0.50–0.69). In order to investigate how the clinical factors jointly affected survival, multivariable proportional Cox model was constructed. When considering all clinical factors together, neoadjuvant radiation also achieved a significantly disease-specific survival benefit (HR, 0.741; 95% CI: 0.646–0.811; *P* = 2.27e-07). Additionally, grade, number of harvested lymph nodes, histological types, tumor size, stage and age also had significant effect on survival (Fig. 2B).

Although Cox model included 23 clinical items that presumably captured most of potential cofounding, some unknown or unmeasured covariates associated with both treatment and outcome still existed. In order to eliminating the effect of unmeasured covariates, we did a sensitivity analysis for the Cox model. Supposing there existed an unmeasured covariant, we changed its parameters (unmeasured confounder HR, prevalence in neoadjuvant group, and prevalence in no radiotherapy group) to see whether we could get similar conclusion for neoadjuvant radiation (Fig. 2C). In most conditions, the unmeasured covariant did not change our conclusion (yellow). The opposite effect of neo-﻿radiotherapy only occurred in some extreme conditions (red). For example, unmeasured confounders whose HR was 2.0 or greater and the prevalence in no radiotherapy group was larger than 0.5. Thus, the beneficial effect of radiotherapy was robust to departures from ignobility.

### Impact of neoadjuvant radiation on survival in different clinical subgroups

Then we grouped patients according to the clinical covariates, and compared the impact of neoadjuvant radiation on survival in different subgroups. Cancer specific survival and Cox model showed that neoadjuvant radiation played significant roles in all stage-related and age-related subgroups, but it did not significantly affect all subgroups of other clinical covariates (see Supplementary Fig. 2A1,D2, Supplementary Table S1).

**Figure 3:**
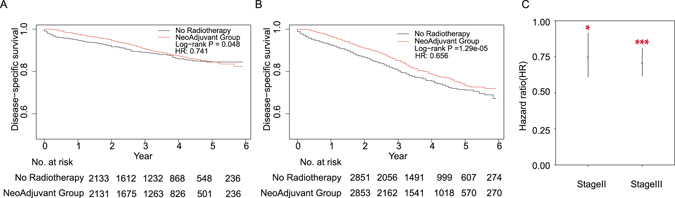
Kaplan-Meier curves for CSS between neoadjuvant radiotherapy and no radiotherapy groups in (**A**) stage II and (**B**) stage III patients. (**C**) HR of neoadjuvant radiotherapy in stage II/III (* means *P* < 0.05; *** means *P* < 0.001).

In both stage II (Fig. 3A) and III (Fig. 3B) patients, the cancer specific survival of neoadjuvant radiation was significantly better than in the no-neoadjuvant group (Log rank *P* = 0.048, 1.29e-05). In stage II patients (n = 4264), the 3-year and 5-year survival rates were 90.6% (95% CI: 89–92.2%) and 84.45% (95% CI: 81.96–87.02%) in neoadjuvant group; 89% (95% CI: 87.4–90.6%), 84.48% (95% CI: 82.29–86.72%) in no-neoadjuvant group. In stage III patients (n = 5704), the 3-year and 5-year survival rates were 85.2% (95% CI: 83.6–86.9%) and 73.3% (95% CI: 70.48–76.2%) in neoadjuvant group; 80.2% (95% CI: 78.4–82.1%), 71.2% (95% CI: 68.67–73.83%) in no-neoadjuvant group. The multivariable Cox model also showed a significant CSS benefits for neoadjuvant radiation in both stage groups (Fig. 3C, 0.741, 0.656; 95% CI: 0.609–0.917, 0.615–0.810; *P* = 0.0213, 3.29e-06).

### Impact of neoadjuvant radiation on survival in younger and older patients

**Figure 4:**
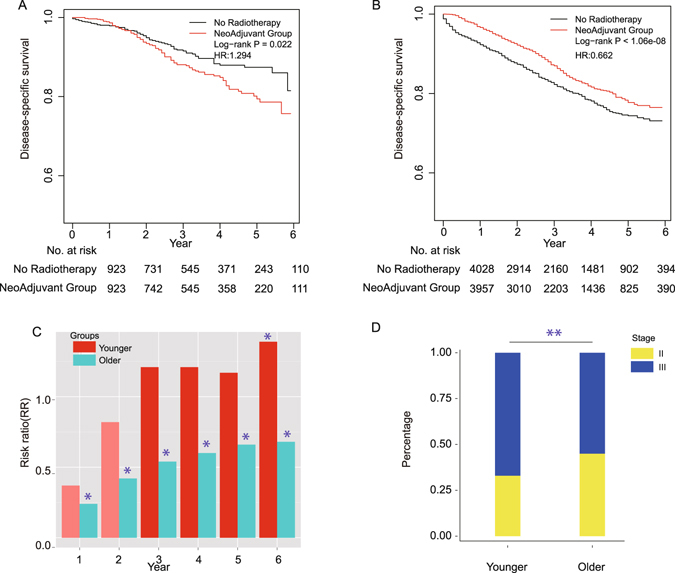
Survival curves, RR and stage distribution in younger and older groups. (**A**) Kaplan-Meier curves for CSS between neoadjuvant (n = 923) and no-neoadjuvant groups (n = 923) in younger patients. (**B**) Kaplan-Meier curves for CSS between neoadjuvant (n = 3957) and no-neoadjuvant groups (n = 4028) in older patients. (**C**) Risk ratio of different year intervals in younger and older groups (* means *P* < 0.05 compared with its own control in younger or older groups). (**D**) Percentage of stage distribution in younger and older groups. (** means *P* < 0.01 compared with older group).

Different from stage-subgroups, neoadjuvant radiation had opposite roles in two subgroups of age. Neoadjuvant radiation improved survival in older patients (*P* < 1.06e-08, see Supplementary Fig. 2E2), but no-neoadjuvant group had better survival than neoadjuvant group in younger patients (*P* = 0.04, see Supplementary Fig. 2E1). In order to confirm the opposite role of neoadjuvant radiation on age, we further excluded the impact of other covariates by using propensity score matching in age-related groups. Since the standardized differences of all covariates were already balanced well in older group (see Supplementary Table S2 right), there was no need to do propensity score matching. One covariate deviated from the balance status in younger group (see Supplementary Table S2 left), therefore we did propensity score matching in younger group and got a new dataset (see Supplementary Table S2 middle part). Survival analysis in this new dataset showed the same trend as the original data (Log rank younger: *P* = 0.022, older: *P* < 1.06e-08, Fig. 4A/B). In younger group (n = 1846), 3-year and 5-year survival rates were 91.5% (95% CI: 89.29–93.81%), 87.4% (95% CI: 84.34–90.58%) in no-neoadjuvant group, versus 88.05% (95% CI: 85.44–90.74%) and 79.42% (95% CI: 75.18–83.39%) in neoadjuvant group. In older group (n = 7985), 3-year and 5-year survival rates were 82.3% (95% CI: 80.8–83.7%), 74.41% (95% CI: 72.37–76.5%) in no-neoadjuvant group, versus 87% (95% CI: 85.6–88.3%) and 77.72% (95% CI: 75.53–79.98%) in neoadjuvant group. Then we adjusted other covariates by Cox proportional model and got the same results: neoadjuvant radiation had a survival benefit in older group (HR: 0.662; 95% CI: 0.586–0.749; *P* = 1.39e-09) while not in younger group (HR: 1.294; 95% CI: 0.946–1.770; *P* = 0.105). Furthermore, we calculated the disease-specific mortality rate in different year interval. For younger group, the RR between neoadjuvant radiation and no-neoadjuvant group was less than 1 in the first two years, but it increased to more than 1 since the 3^rd^-year. Neoadjuvant radiation was significantly associated with increased disease-specific mortality in the 6^th^-years. However, in older group neoadjuvant radiation was associated with reduced disease-specific mortality significantly (RR < 1) (Fig. 4C). We noted that cancer specific survival and mortality rate after 3 years were slightly different from that within 3 years (Figs 2A, 3A,B and 4C). To investigate this difference thoroughly, we split patients into short-term (<3 years) group and long-term (> = 3years) group. Result showed that neoadjuvant radiation improved short-term survival in both younger and older patients, especially for older patients. However, there was no long-term survival benefit in older group and younger group, especially for younger group (see Supplementary Fig. 3). Additionally, based on 3^th^ year survival we calculated the 5 year conditional survival which was a kind of more accurate quantification of prognosis for long-term survivors^7^. Result showed that neoadjuvant group has poorer 5 year conditional survival than no-neoadjuvant group (28.43% vs. 30.05%) in younger patients and neoadjuvant group had better 5 year conditional survival than no-neoadjuvant group (26.62% vs. 27.08%) in older patients. The underlying reasons for this long term and short term difference might be variable. Previous study shows that in younger patients who develop the local recurrence die faster than older patients^45^. One possible reason for this might be that in younger patients neoadjuvant radiation group had higher local recurrence rate than no-neoadjuvant radiation group. So in the long term when both groups developed distant metastases and died of disease, neoadjuvant group had poorer survival. However, in older group, although neoadjuvant radiation had better ability to control the local recurrence in the short term, in the long term they had similar local recurrence rate and survival rate.

**Figure 5:**
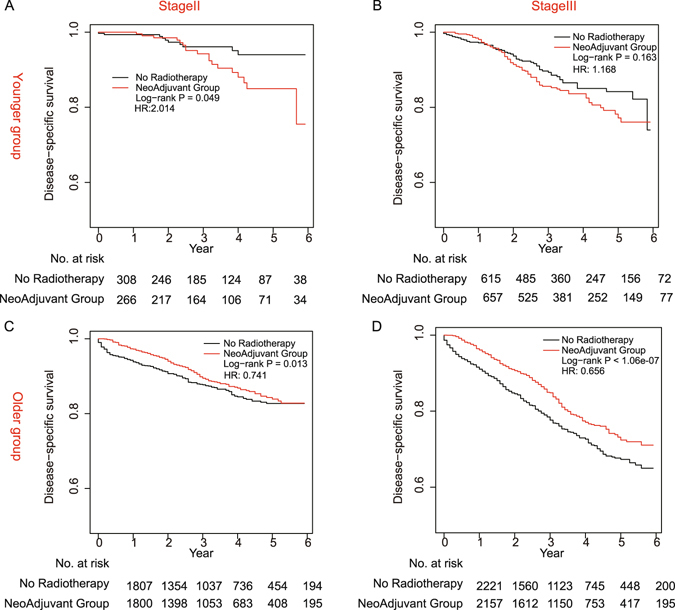
Kaplan-Meier curves for CSS between adjuvant radiotherapy and no radiotherapy groups in four patient subgroups. (**A**) CSS in younger stageII group (n = 266). (**B**) CSS in younger stage III groups (n = 657). (**C**) CSS in older stageII group (n = 1800). (**D**) CSS in older stage III groups (n = 2157).

Additionally, we analyzed the difference of every covariate between two age groups. Fisher’s exact test showed that there were significantly different distributions in gender, tumor number, stage, tumor size, lymph nodes resected, histological type and grade between younger group and older group (see Supplementary Table S3), which in same covariates was consistent with previous research^19, 23^. Tumors of younger patients tended to be in later stage (Fig. 4D). Since both age and stage were significantly associated with the benefit of neoadjuvant radiation, we further split patients by age and stage to investigate their combination effects. Survival curves were constructed for four subgroups. In younger group (Fig. 5A,B), neoadjuvant radiation did not have survival benefit no matter of the stages, and this trend was worse in stage II (Log-rank *P* = 0.049, *P* = 0.163). In older group (Fig. 5C,D), neoadjuvant radiation have survival benefit no matter of the stages, and this trend was better in stage III (Log-rank *P* < 1.06e-07, *P* = 0.013). Finally, we calculated the neoadjuvant radiation’s HRs in four groups (Table 2). Result indicated that neoadjuvant radiation played different roles in different sub-groups. Patients in later stage of disease and in older group tended to obtain more survival benefits from the neoadjuvant radiation.

**Table 2:** HR and *P* value in different age and stage groups.

	Hazard ratio (Stage II)	95% CI	*P*	Hazard ratio (Stage III)	95% CI	*P*
< = 50	2.014	(0.9032–4.490)	0.0871	1.168	(0.8298–1.6461)	0.3723
>50	0.741	(0.5993–0.9168)	0.0058	0.656	(0.5646–0.7642)	5.26e-08

## Discussion

In the present study, we used propensity score matching to get a large baseline covariates balanced cohort of rectal cancer, and investigated effectiveness of neoadjuvant radiotherapy on survival. And Cox model confirmed previous reported significant prognostic factors (Fig. 2B, see Supplementary Table S4), such as tumor size^31^, histological type^32^, differentiation^31^, age^6^, number of harvest lymph node^33, 34^. In all results, neoadjuvant radiation had cancer specific survival benefit not only in the whole cohort but also in different stage subgroups. Previous studies shows that stage III patients tend to have more survival benefits from neoadjuvant/adjuvant therapy compared with stage II^15, 26^, which was consistent with our result. However, neoadjuvant radiotherapy had opposite survival benefit in younger and older groups.

We analyzed the effectiveness of neoadjuvant radiotherapy according to age. Neoadjuvant radiotherapy in younger group showed increasing survival risk. From our disease-specific mortality analysis, neoadjuvant radiotherapy was associated with increase disease-specific mortality starting from 3 years (Fig. 4C). Recently the incidence of CRC tends to be increasing among adults younger than 50 years^19^, and previous studies shows that rectal and distal colon are identified as predilection location in younger CRC patients^19, 23^. Age is an important factor when considering the treatment for patients^24^. Therefore they suggests starting rectal cancer screening before age 50^46^. These studies showed that more attention has been paid to younger patients. Our results on neoadjuvant radiotherapy also suggested that younger patients should be distinguished from older patients when making treatment decision.

Since this work was based on population-based cancer registry, there exist some limitations. Population study has limited internal validity compared with randomized controlled trials (RCTs), exists many confounding variables and study design is limited^47^. Many pitfalls exist in population-based state cancer registry data, such as: multiple cancer diagnoses, duplicate reports, reporting delays, misclassification of race/ethnicity, and pitfalls in estimations of cancer incidence rates, etc^48^. So analysis results based on population-based study are prone to multiple biases. In this study, we adopted propensity score matching method to mitigate some potential sources of biases. After matching, sensitivity analysis showed (Fig. 2C) that unmeasured confounders did not influence our conclusions except their hazard ratio higher than 1.3. Such result was similar with previous study^27, 41^ and indicated the robustness of our results.

Although many covariates had being considered in our analysis, some important clinical factors and prognostic biomarkers were not included due to the lack of information in SEER database, such as: MSI, expression of biomarkers, local recurrence, details of radiotherapy such as dose, field, radiation treatment technique etc. Previous study shows that different age group (< = 50, >50) has different prognostic biomarkers, such as the *PRL*, *RBM3*, *Wrap53*, *p53* and DNA status. *PRL* expression was negatively related with CSS in young CRC patients^6^. Receiving neoadjuvant radiotherapy, strong PRL expression and *FXYD-3* expression is unfavorable prognostic in patients^49, 50^. Some other genes has been assessed the predictor’s role in neoadjuvant radiochemotherapy such as *p21*, *VEGF* etc^51^. Besides, Previous research finds MSI is very important good marker of prognosis in stage II/III CRC^52^. A former study showed that MSI could not predict therapeutic response to neoadjuvant radiotherapy^53^. However, this relationship has not fully demonstrated. In younger and older rectal patient groups, there is different percentage of MSI and different studies has different MSI percentage in younger and older rectal patients^54, 55^. Besides, in younger patients, 2–7% MSI are associated with Lynch syndrome^56^. Apart from these biomarkers, local recurrence is also an important prognostic factor and clinical indicator for quality of neoadjuvant treatment in rectal cancer. Local recurrence is an painful event for rectal cancer patients and limited success is achieved by further salvage surgery treatment^57^. A study of cohort who underwent R0 or R1 resections showed that patients die faster if diagnosing with local recurrence and local recurrence is the single most important indicator for reduced survival in this study^45^. Neoadjuvant radiation can improve local control for rectal cancer conclusively^15, 58^. Different neoadjuvant regimens followed by different surgery has different local control rate, and the standard care for stage II and stage III rectal cancer is neoadjuvant radiotherapy followed by Total Mesorectal Excision (TME), which leads to a decrease in local recurrence rates to 6%^25^. Other study shows that short-course preoperative radiotherapy can reduce local recurrence and can maintain this ability when combined with TME^59^. A randomized study showed that short-course and long-course neoadjuvant radiotherapy are not statistically significant in local recurrence rate^13^.

In future, we would like to do more studies about neoadjuvant radiotherapy. Firstly, results obtained from our population-based study are needed to be validated by more comprehensive and accurate data. Therefore, we expect to analyze data from randomized clinical trials via scientific collaboration. Secondly, we would like to explore the molecular-level differences between younger and older patients before and after neoadjuvant radiation. This may help our understanding of the different effects of neoadjuvant radiotherapy according to age. Thirdly, we will investigate the challenging problem of predicting response to neoadjuvant radiotherapy. We hope find an optimal radiotherapy plan for individual patient by combining clinical factors and molecular markers.

In conclusion, our study provided new insights on the neoadjuvant radiation in rectal cancer, especially for the younger patients; And provided reliable information to guide future knowledge translation of neoadjuvant radiation in younger patients, especially for long term ineffectiveness of neoadjuvant radiation in younger patients. Additionally, our results provided evidence of effectiveness of neoadjuvant radiotherapy for older patients, especially in short term cancer specific survival benefit.

## Electronic supplementary material

Supplementary information

Supplementary Table 1

Supplementary Table 2

Supplementary Table 3

Supplementary Table 4
