# Supplementary material for: Population-based study of effectiveness of neoadjuvant radiotherapy on survival in US rectal cancer patients according to age
Source: Sci Rep. 2017 Jun 14;7:3471. doi: 10.1038/s41598-017-02992-7 (PMC5471198; doi:10.1038/s41598-017-02992-7)
Supplement: Supplementary file 1 — Supplementary information [file 41598_2017_2992_MOESM1_ESM.pdf]

Population-based study of effectiveness of neoadjuvant radiotherapy on survival in US rectal cancer patients according to age

Leilei Wu[1],Shichao Pang[4],Qianlan Yao[1], Chen Jian[5], Ping Lin[2], FangyouminFeng[2,6], Hong Li[2,*],Yixue Li[1,2,3,*]

[1] School of Life Sciences and Biotechnology, Shanghai Jiao Tong University, Shanghai, 200240, China;

[2] CAS Key Laboratory for Computational Biology, CAS-MPG Partner Institute for Computational Biology, Shanghai Institute for Biological Sciences, Chinese Academy of Sciences, Shanghai, 200031, China;

[3] Collaborative Innovation Center of Genetics and Development, Fudan University, Shanghai, 200433, China;

[4] Department of Statistics, School of Mathematical Sciences, Shanghai Jiao Tong University, Shanghai,200240, China;

[5] Department of General Surgery, Shanghai General Hospital, Shanghai Jiao Tong University School of Medicine, songjiang district, 201600, shanghai;

[6] School of Life Science and Technology, ShanghaiTech University, Shanghai 201210, China;

* To whom correspondence should be addressed. Tel: 86-21-54920079; Fax: 86-21-54920079; Email: yxli@sibs.ac.cn.

Correspondence may also be addressed to Hong Li. Tel: 86-21-54920079; Fax: 86-21-54920079; Email: lihong01@sibs.ac.cn

SUPPLEMENTARY TABLE, FIGURE LEGENDS AND ABERRATION

Supplementary Table S1: HR and 95% CI of neoadjuvant radiation in subgroups of significant covariates.

Supplementary Table S2: Comparison of clinical characteristics between patients with or without neoadjuvant radiotherapy in younger.

Supplementary Table S3: Clinic pathological items of rectum cancer patients according to age groups.

Supplementary Table S4: Multivariate survival analysis of prognostic factors in stage II and III patients, respectively.

Supplementary Figure 1: Distribution and incidence of rectal cancer by age. A): Distribution in different age interval in our propensity score matched cohort. B): Percentage of rectal cancer patients in younger (<=50) and older groups (>50). C): Trend of incidence rates (per 100,000) of rectal cancer in younger group (read solid line) and older groups (blue dotted line) from 1992 to 2012.

Supplementary Figure 2: Disease-specific Survival curves of neoadjuvant radiation in all significant covirates group. A1~A4: 5 year disease specific survival curves between neoajuvant radiation and control in four different grade groups(A1:well;A2:moderately;A3:Poorly;A4: undifferentiated). B1~B2: 5 year disease specific survival curves between neoajuvant radiation and control in different lymph node resected groups(B1:>12;B2<=12). C1~C2: 5 year disease specific survival curves between neoajuvant radiation and control in different histological type groups(C1:Mucinous;C2: Non-mucinous). D1~D2: 5 year disease specific survival curves between neoajuvant radiation and control in different tumor size groups (D1:>5;C2:<=5). 5 year disease specific survival curves between neoajuvant radiation and control in different age groups((E1<=50;E2>50).

Supplementary Figure 3: Cancer-specific Survival curves of neoadjuvant radiation for short-term(<=3, ST) and long term(>3, LT) group in younger and older patients. A) Cancer-specific survival for ST in younger patients. B) Cancer-specific survival for ST in older patients. C) Cancer-specific survival for LT in younger patients. D) Cancer-specific survival for LT in older patients. All figures also report HR of neoadjuvant radiation in different situation.

Aberration

SEER	Surveillance, Epidemiology, and End Result
MSI	MicroSatellite Instability
CRC	ColoRectal Cancer
S.D	Standardized Difference

CSS	Cancer-Specific Survival
RCTs	Randomized Controlled Trials
TME	Total Mesorectal Excision
HR	Hazard Ratio
RR	Risk Ratio

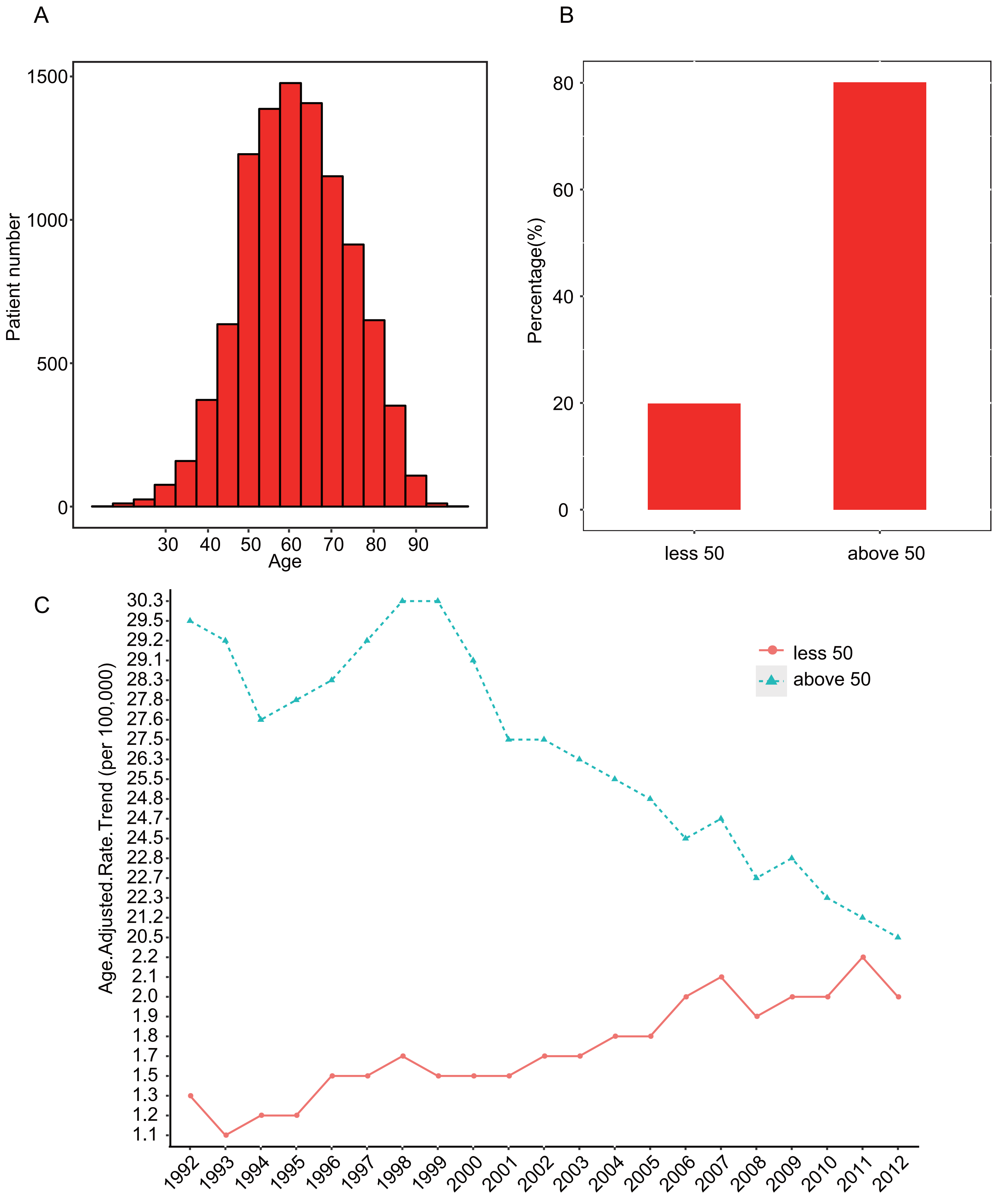
A
1500
1000
500
0
Patient number
30
40
50
60
70
80
90
Age
B
80
60
40
20
0
Percentage(%)
less 50
above 50
C
Age.Adjusted.Rate.Trend (per 100,000)
30.3
29.5
29.2
29.1
28.3
27.8
27.6
27.5
26.3
25.5
24.8
24.7
24.5
22.8
22.7
22.3
21.2
20.5
2.2
2.1
2.0
1.9
1.8
1.7
1.5
1.3
1.2
1.1
less 50
above 50
1992
1993
1994
1995
1996
1997
1998
1999
2000
2001
2002
2003
2004
2005
2006
2007
2008
2009
2010
2011
2012

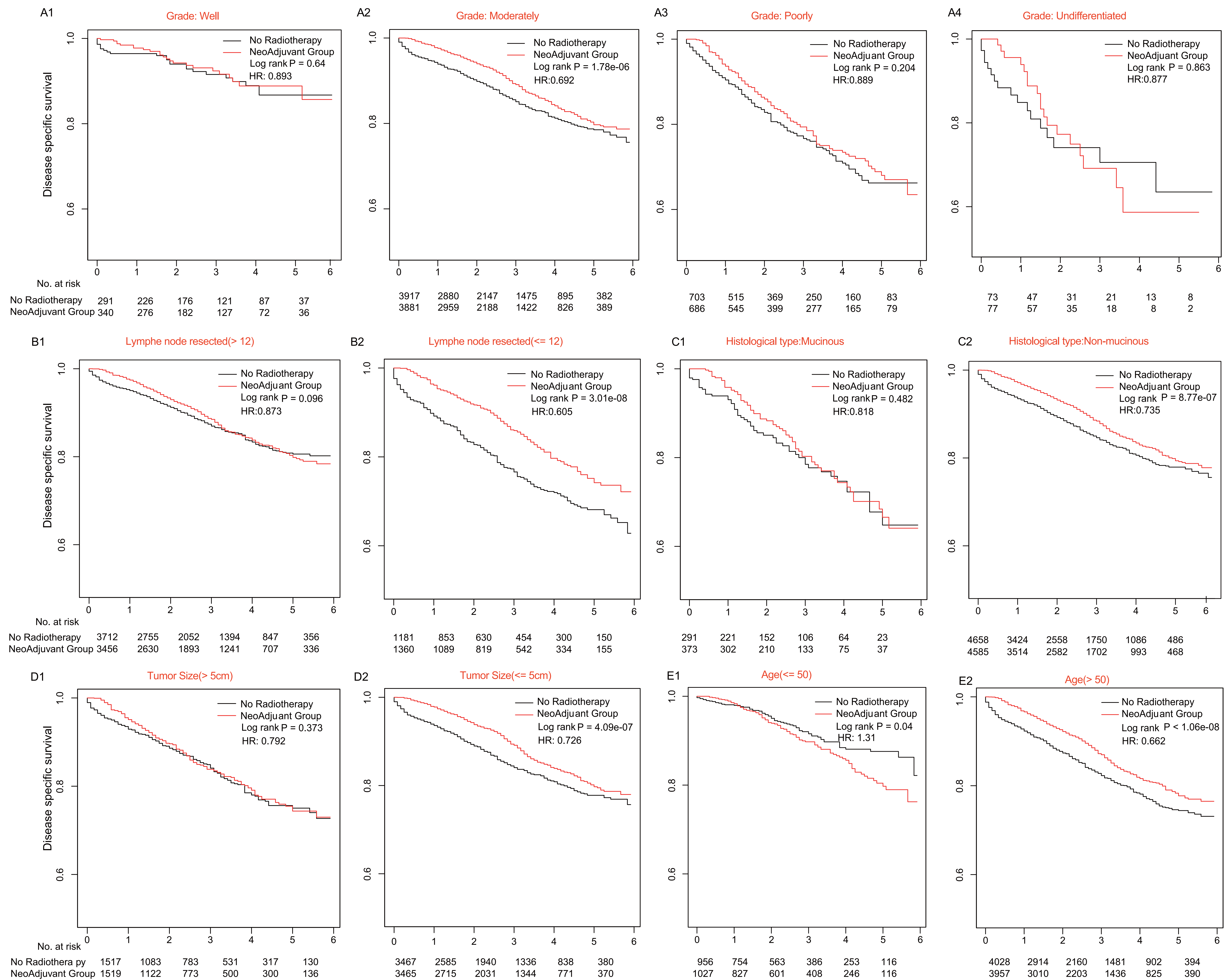
A1
Grade: Well
No Radiotherapy
NeoAdjuvant Group
Log rank P = 0.64
HR: 0.893
Disease specific survival
No. at risk
No Radiotherapy 291 226 176 121 87 37
NeoAdjuvant Group 340 276 182 127 72 36
A2
Grade: Moderately
No Radiotherapy
NeoAdjuvant Group
Log rank P = 1.78e-06
HR:0.692
3917 2880 2147 1475 895 382
3881 2959 2188 1422 826 389
A3
Grade: Poorly
No Radiotherapy
NeoAdjuvant Group
Log rank P = 0.204
HR:0.889
703 515 369 250 160 83
686 545 399 277 165 79
A4
Grade: Undifferentiated
No Radiotherapy
NeoAdjuvant Group
Log rank P = 0.863
HR:0.877
73 47 31 21 13 8
77 57 35 18 8 2
B1
Lymphe node resected(> 12)
No Radiotherapy
NeoAdjuvant Group
Log rank P = 0.096
HR:0.873
Disease specific survival
No. at risk
No Radiotherapy 3712 2755 2052 1394 847 356
NeoAdjuvant Group 3456 2630 1893 1241 707 336
B2
Lymphe node resected(<= 12)
No Radiotherapy
NeoAdjuvant Group
Log rank P = 3.01e-08
HR:0.605
1181 853 630 454 300 150
1360 1089 819 542 334 155
C1
Histological type:Mucinous
No Radiotherapy
NeoAdjuvant Group
Log rank P = 0.482
HR:0.818
291 221 152 106 64 23
373 302 210 133 75 37
C2
Histological type:Non-mucinous
No Radiotherapy
NeoAdjuvant Group
Log rank P = 8.77e-07
HR:0.735
4658 3424 2558 1750 1086 486
4585 3514 2582 1702 993 468
D1
Tumor Size(> 5cm)
No Radiotherapy
NeoAdjuvant Group
Log rank P = 0.373
HR: 0.792
Disease specific survival
No. at risk
No Radiothera py 1517 1083 783 531 317 130
NeoAdjuvant Group 1519 1122 773 500 300 136
D2
Tumor Size(<= 5cm)
No Radiotherapy
NeoAdjuvant Group
Log rank P = 4.09e-07
HR: 0.726
3467 2585 1940 1336 838 380
3465 2715 2031 1344 771 370
E1
Age(<= 50)
No Radiotherapy
NeoAdjuvant Group
Log rank P = 0.04
HR: 1.31
956 754 563 386 253 116
1027 827 601 408 246 116
E2
Age(> 50)
No Radiotherapy
NeoAdjuvant Group
Log rank P < 1.06e-08
HR: 0.662
4028 2914 2160 1481 902 394
3957 3010 2203 1436 825 390

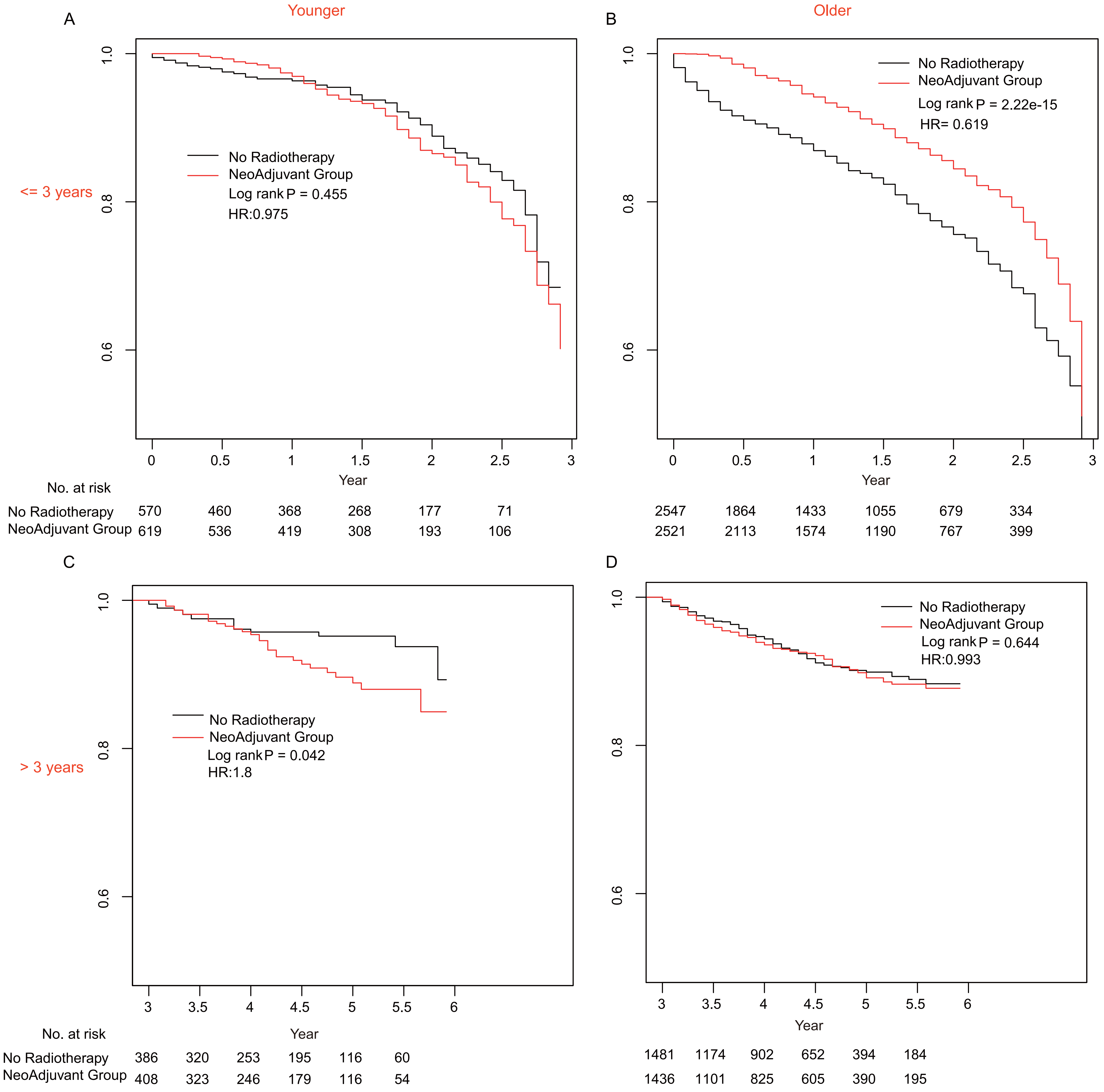
A
Younger
B
Older
<= 3 years
> 3 years
No Radiotherapy
NeoAdjuvant Group
Log rank P = 0.455
HR:0.975
Log rank P = 2.22e-15
HR= 0.619
Log rank P = 0.042
HR:1.8
Log rank P = 0.644
HR:0.993
1.0
0.8
0.6
0 0.5 1 1.5 2 2.5 3
3 3.5 4 4.5 5 5.5 6
Year
No. at risk
No Radiotherapy 570 460 368 268 177 71
NeoAdjuvant Group 619 536 419 308 193 106
2547 1864 1433 1055 679 334
2521 2113 1574 1190 767 399
C
D
No Radiotherapy 386 320 253 195 116 60
NeoAdjuvant Group 408 323 246 179 116 54
1481 1174 902 652 394 184
1436 1101 825 605 390 195
